# Sarcoendoplasmic reticulum calcium ATPase is an essential and druggable *lipid-dependent* ion pump in *Toxoplasma gondii*

**DOI:** 10.1038/s42003-025-08058-z

**Published:** 2025-05-06

**Authors:** Rosalba Cruz-Mirón, Namita Pandey, Dimitrios Alexandros Katelas, Arunakar Kuchipudi, Dharmarajan Sriram, Aditi Gangopadhyay, Soumyananda Chakraborti, Ratnesh Kumar Srivastav, Nishith Gupta

**Affiliations:** 1 Intracellular Parasite Education and Research Labs (iPEARL), Department of Biological Sciences, Birla Institute of Technology and Science, Pilani (BITS Pilani), Hyderabad, India; 2 Department of Molecular Parasitology, Faculty of Life Sciences, Humboldt University, Berlin, Germany; 3 Department of Pharmacy, Birla Institute of Technology and Science, Pilani (BITS Pilani), Hyderabad, India; 4 Department of Chemical Technology, University of Calcutta, Kolkata, India; 5 Department of Biological Sciences, Birla Institute of Technology and Science, Pilani (BITS Pilani), Hyderabad, India

**Keywords:** Parasite biology, Molecular modelling

## Abstract

*Toxoplasma gondii* is a common intracellular pathogenic protist causing acute and chronic infections in many warm-blooded organisms. Calcium homeostasis is pivotal for its asexual reproduction in mammalian host cells, and sarcoendoplasmic reticulum calcium-ATPase (SERCA) is considered vital for maintaining ion homeostasis within the parasite. This work studied the physiological relevance, structure-function relationship, mechanism, and therapeutic value of SERCA in the acutely-infectious tachyzoite stage of *T. gondii*. A conditional depletion of SERCA, located in the endoplasmic reticulum, by auxin-inducible degradation is lethal for the parasite due to severe defects in its replication, gliding motility, and invasion. The observed phenotypes are caused by dysregulated calcium ion homeostasis and microneme secretion in the absence of *Tg*SERCA. Furthermore, ectopic expression of *Tg*SERCA restored the lytic cycle of a phosphatidylthreonine-null and phosphatidylserine-enriched mutant with perturbed calcium homeostasis, motility and invasion. These lipids are expressed in the parasite ER, co-localizing with *Tg*SERCA. Last but not least, the structure-function modeling and ligand docking of *Tg*SERCA with a library comprising >5000 chemicals identified two compounds (RB-15, NR-301) that inhibited the lytic cycle by affecting the tachyzoite locomotion, invasion, microneme discharge, and calcium levels. In conclusion, we demonstrate *Tg*SERCA as an indispensable *lipid-assisted* calcium pump in *T. gondii* and report small molecules with therapeutic potential against toxoplasmosis.

## Abstract (web summary)

*Toxoplasma gondii* depends on SERCA for calcium homeostasis and its lytic cycle. Conditional knockdown of SERCA is lethal for the parasite and two novel inhibitors (RB-15, NR-301) show therapeutic potential against toxoplasmosis.

## Introduction

*Toxoplasma gondii* is an obligate intracellular parasitic protist capable of infecting and reproducing in several vertebrate organisms, including humans. Approximately one-third of the world population has been exposed to this prevalent pathogen, which usually persists in the encysted dormant form throughout the life of an infected host^1,2^. Its highly infectious tachyzoite stage undergoes recurrent lytic cycles in several types of nucleated host cells. Tachyzoites are motile cells with a polarized body designed to actively invade and replicate in a repertoire of host cells. Once intracellular, they proliferate every 8–10 h by endodyogeny (forming two progeny within a mother cell)^3^ and then egress by lysing host cells, leading to tissue necrosis and acute toxoplasmosis. Several cascades, including calcium signaling, regulate the lytic cycle in a spatiotemporal manner^4^.

Calcium homeostasis and signaling in *T. gondii* involve several organelles, such as the endoplasmic reticulum (ER), Golgi network, mitochondrion, acidocalcisomes, and a plant-like vacuole^4^. A regulated release of calcium to the cytosol and reuptake into an internal store or exit into the extracellular milieu is vital for cellular ion homeostasis. In mammalian cells, the SERCA family proteins maintain calcium levels by catalyzing the ATP-dependent translocation of calcium from the cytoplasm into the ER lumen, thereby restoring the resting-state ion level^5^. Similarly, ER is a significant store housing ion pumps and channels in *T. gondii* that, under different conditions, regulate the influx and efflux of calcium to/from the cytosol^6^. SERCA is one such protein, first reported by Nagamune K et al.^7^. Its Inhibition by thapsigargin, a human SERCA inhibitor, results in elevated cytosolic calcium, which in turn stimulates exocytosis of micronemes, a specialized secretory organelle in *T. gondii*^8,9^. Secretion of micronemes triggers the gliding motility in tachyzoites^10^, which drives the host-cell invasion and parasite egress.

Parasite motility is regulated by calcium signaling *via* activation of the ‘glideosome’—an actomyosin motor^11^. The micronemal proteins discharged by tachyzoites bind to the matrix or host-cell membrane, providing traction for gliding motility. The secretion of micronemes can be induced by incubating tachyzoites with A23187 (an ionophore) and inhibited by BAPTA-AM (a calcium chelator)^12^. Ionophores, such as A23187, can bypass the protein-regulated release of calcium and rapidly increase its cytosolic amount, causing parasites to egress from host cells^13^. Although the role of calcium in parasite replication has not been determined, it is an essential regulator of mitotic division in mammalian cells^14,15^. Consequently, SERCA is expected to support major events during the lytic cycle of *T. gondii*, though its functional relevance, structural characteristics, and therapeutic potential remain poorly understood.

In mammalian cells, the phospholipid milieu, among other factors, can also influence SERCA function. Specifically, perturbation of the ER membrane lipids dysregulates the calcium homeostasis^16,17^. A genetic perturbation or inhibition of phospholipid synthesis in *T. gondii* is often associated with impaired replication due to defective membrane biogenesis^18–23^. In recent years, however, a few anionic phospholipids, namely phosphatidic acid (PtdOH), phosphatidylinositol (PtdIns), phosphatidylserine (PtdSer), and phosphatidylthreonine (PtdThr), have been suggested to facilitate the gliding motility, invasion, egress, calcium homeostasis, and micronemal exocytosis during tachyzoite infection^19,24–32^. In the context of this work, we have previously demonstrated that PtdThr is synthesized in the ER of tachyzoites, and the deletion of PtdThr synthase (PTS) compromises calcium storage/discharge, resulting in poor motility, egress, and invasion, but not replication^27–29,33^. The underlying basis of this intriguing finding is not clear. Our findings, herein, establish SERCA as a critical *lipid-dependent* protein with a significant therapeutic potential.

## Results

### Knockdown of *Tg*SERCA by auxin-inducible degron in tachyzoites

**Fig. 1:**
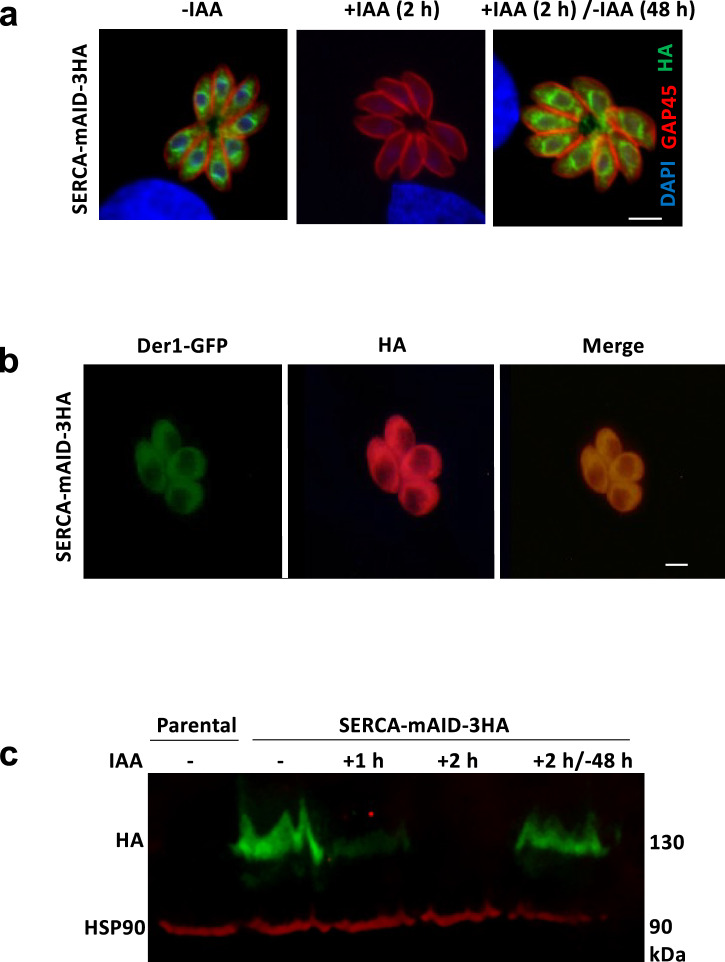
Conditional depletion of SERCA in tachyzoites by 3’-genomic tagging with a mini auxin-inducible degron. **a** Immunofluorescent images visualizing SERCA’s localization, depletion, and recovery in response to IAA (Scale, 5 μm). Parasite cultures, with or without IAA incubation (500 μM), were immunostained for the expression of HA and *Tg*GAP45. **b** Co-localization of SERCA-mAID-3HA with an ER marker (Der1-GFP) after transfection of the mutant with a plasmid expressing Der1-GFP (24 h post-infection). Immunostaining was performed using the αHA and αGFP antibodies (Scale, 5 μm). **c** Immunoblot displaying IAA-dependent expression of SERCA-mAID-3HA in the mutant. Parasites (±500 μM IAA) were subjected to SDS-PAGE and western blot analysis using the αHA and α*Tg*HSP90 antibodies.

To determine the importance of SERCA for the lytic cycle, we engineered a conditional mutant by 3’-tagging of its predicted gene (TGGT1_230420, www.ToxoDB.org) with a mini auxin-inducible degron (mAID, Fig. S1). This system allows proteasomal degradation of mAID-fused proteins by culturing cells with indole-3-acetic acid (IAA)^34^. The crossover-mediated integration events at the target locus were confirmed by genomic screening PCR using recombination-specific primers (Fig. S1). As envisaged, the clonal SERCA-mAID-3HA mutant delivered amplicons of the expected size (PCR1 and PCR2), unlike the parental control strain. Conversely, genomic PCR using locus-specific primers (PCR3) amplified a product in the parental strain, but not in the mutant. Immunofluorescent staining revealed the expression of SERCA-mAID-3HA fusion protein in the perinuclear region (Fig. 1a), co-localizing with an ER marker (Fig. 1b) and confirming previous studies^8,35^. It became undetectable in parasites cultured with IAA for 2 h (Fig. 1a). Immunoblot revealed a protein band of the expected size (~130-kDa) and confirmed a rapid depletion of SERCA within 2 h of auxin treatment (Fig. 1c). The process was also reversible, as judged by the reappearance of SERCA after the withdrawal of IAA. These data validated a conditional SERCA-mAID-3HA mutant for downstream assays.

### *Tg*SERCA is essential for the lytic cycle of *T. gondii*

**Fig. 2:**
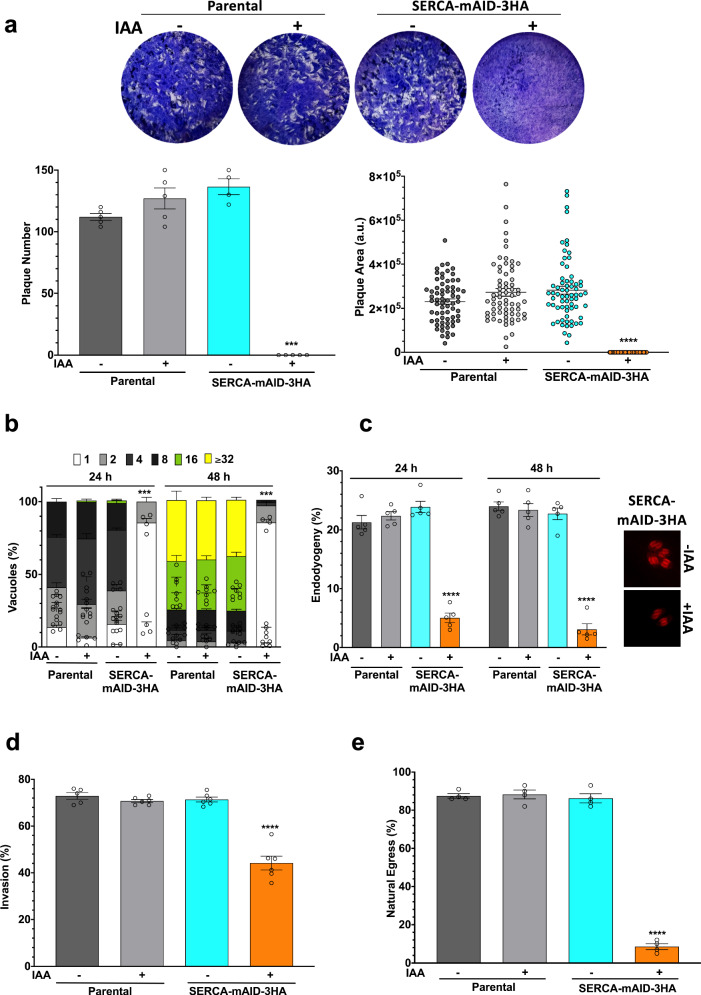
IAA-induced knockdown of SERCA impairs the replication, invasion, and natural egress of *T. gondii.* **a** Plaques produced by SERCA-mAID-3HA and parental strains. Crystal violet-stained images show plaques (white) formed after sequential lytic cycles of tachyzoites in HFF monolayers (blue). Quantitative evaluation of plaque size in arbitrary units (a. u.) and numbers are shown in graph. No plaques were detectable upon SERCA depletion in the mutant (500 μM IAA, 7 d). **b** The replication rates of specified strains. *Tg*GAP45-stained tachyzoites within their PV were quantified to gauge the proliferation. **c** The budding of progeny within a parent cell (endodyogeny), as scored by *Tg*IMC3-positive parasites. The inset images show daughter cell formation in the SERCA-mAID-3HA strain (±IAA). **d**, **e** Invasion and natural egress. Parasites were pre-cultured with 500 μM IAA (18 h) to make the inoculum. The (**a**–**e**) show data from *n* = 4 or 5 assays (means ± S.E.).

Our follow-up work deployed the SERCA-mAID-3HA strain to examine the impact of SERCA depletion on tachyzoites. We first performed plaque assays, which recapitulate the successive lytic cycles and the parasite fitness in standard cultures (Fig. 2a). The parental strain displayed normal growth irrespective of IAA exposure, whereas the mutant failed to produce detectable plaques upon loss of SERCA (Fig. 2a). We next quantified the replication rates by enumerating tachyzoites multiplying within parasitophorous vacuoles (PV) in early (24 h) and late (48 h) cultures (Fig. 2b). As predicted, the parental strain was not affected by IAA and yielded a higher fraction of larger PVs at 24 h (2-8 parasites) and 48 h (16-≥32 parasites). Conversely, most vacuoles of the IAA-treated SERCA-mAID-3HA strain harbored only 1-2 parasites, signifying a strong defect in proliferation. Further evaluation of cell division showed the impairment of endodyogeny in the SERCA-mAID-3HA strain treated with IAA (Fig. 2c with representative images of the mutant). Approximately 20–25% of parasites displayed budding in control cultures (-IAA), which was notably reduced upon SERCA knockdown. The invasion was also significantly reduced after auxin-induced SERCA depletion (Fig. 2d). Impairment of replication and invasion resulted in poor natural egress of the mutant (Fig. 2e). These results together demonstrated a critical requirement of SERCA for the lytic cycle.

### *Tg*SERCA facilitates the parasite motility, micronemal secretion, and calcium homeostasis

**Fig. 3:**
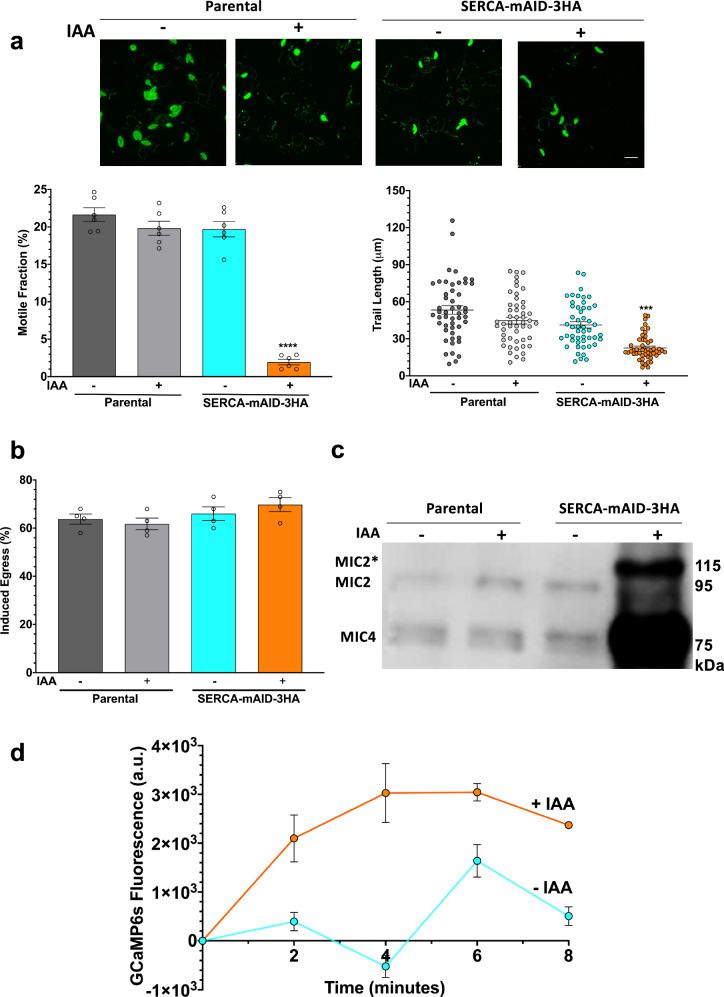
SERCA facilitates the parasite motility, microneme secretion, and calcium homeostasis in *T. gondii.* **a** Gliding motility of the SERCA-mAID-3HA and parental strains. *Tg*SAG1-stained parasites were scored for the motile fraction and trail length (*n* = 6 experiments, means ± S.E.). **b** Ionophore (A23187)-induced egress (*n* = 4 assays, means ± S.E.). **c** Western blot of the parasite-secreted proteins using α*Tg*MIC2 and α*Tg*MIC4 antibodies. Detection of pre-processed MIC proteins in the discharge is likely due to inadvertent lysis of tachyzoites. **d** GCaMP6s-based quantification of calcium levels in tachyzoites. The SERCA-mAID-3HA mutant expressing GCaMP6s was cultured (±500 μM IAA, 18 h), parasites were treated with 5 μM A23187, and the relative fluorescence units (RFU) were measured (*n* = 3 assays, means ± S.E.). Note that the graph includes the data points; however, they are not visible due to the large Y-axis. The original values used for this graph can be accessed in the Supplemental Data file.

Next, we focused on determining the basis of observed phenotypes in the SERCA mutant. A surge in cytosolic calcium at the end of parasite replication coincides with the secretion of microneme proteins and activation of gliding motility^36–38^, which drives the parasite to exit decrepit host cells and invade adjacent cells. We compared the SERCA-mAID-3HA mutant to the parental strain for these phenotypes (Fig. 3). Indeed, the parasite’s motile fraction and trail length were remarkably reduced in the presence of IAA (Fig. 3a). Besides, the egress phenotype of the SERCA-deprived strain was fully restored by a calcium ionophore (A23187, Fig. 3b), which is known to trigger calcium influx into parasite cytosol from intracellular and extracellular sources^12,13^.

An increase in cytosolic calcium of tachyzoites leads to the discharge of micronemal proteins, some of which (e.g., MIC2) are critical for parasite motility^39^. Hence, we performed a micronemal secretion assay to deduce the subcellular events facilitated by SERCA (Fig. 3c). As expected, the parental strain secreted processed MIC2 (~95-kDa) and MIC4 (~75-kDA) proteins irrespective of IAA exposure. In the absence of IAA, the SERCA-mAID-3HA strain mimicked the parental parasites. However, treatment with the auxin caused an accumulation of pre-processed MIC2 (~115-kDa, MIC2*) and MIC4, while the release of mature protein declined upon SERCA depletion (Fig. 3c). Finally, we examined the SERCA-mAID-3HA mutant for its potential to restore the cytosolic calcium after A23187-induced discharge (Fig. 3d). In this regard, we expressed GCaMP6s—a gene-encoded calcium sensor^40^, in the SERCA-mAID-3HA strain. Quantification of GCaMP6s revealed elevated calcium in the ionophore-treated SERCA-depleted parasites (Fig. 3d), indicating impaired refill of calcium into the ER. Our phenotyping assays suggest that a perturbation of calcium in the absence of SERCA affects the micronemal secretion, which impairs the parasite locomotion and motility-dependent events in *T. gondii*.

### *Tg*SERCA displays a conserved topology but a flexible ATP-binding pocket

**Fig. 4:**
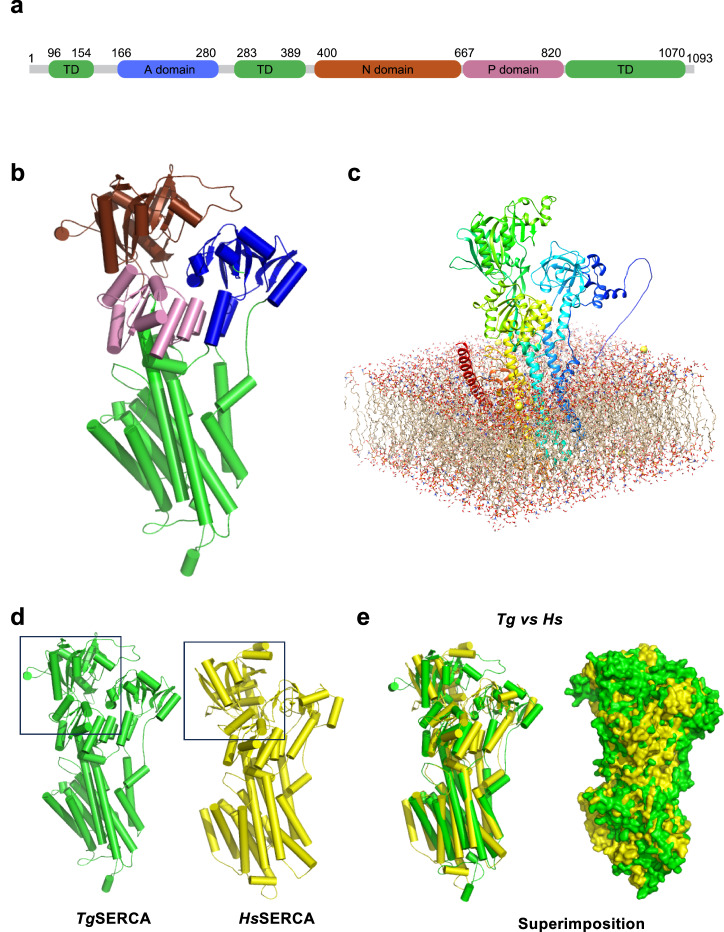
Structural comparison of *Tg*SERCA with *Hs*SERCA2a. **a** The primary structure of *Tg*SERCA featuring domains and transmembrane helices. Cytoplasmic domains are depicted as blue (**A** domain), brown (**N** domain), and pink (**P** domain) boxes. **b** A homology model of *Tg*SERCA. The domain arrangement of *Tg*SERCA resembles its human counterpart. **c** The membrane-bound illustration of *Tg*SERCA with transmembrane helices (M1–M10) embedded in the lipid bilayer. **d**, **e** Structural superimposition of *Tg*SERCA with *Hs*SERCA2a, emphasizing their resemblance.

The SERCA family proteins comprise cytoplasmic and membrane-bound domains (Fig. 4a). Equally, *Tg*SERCA harbors three transmembrane and three cytoplasmic domains (**A**, actuator; **N**, nucleotide binding; **P**, phosphorylation). **A** is flanked by two transmembrane domains. The **N** and **P** domains are juxtaposed delimited by a transmembrane domain. Notably, all three cytoplasmic domains of *Tg*SERCA are larger than those of human and *Plasmodium* counterparts. The absence of a SERCA structure in the PDB for any parasite motivated us to model *Tg*SERCA using its human ortholog, SERCA2a (PDB: 7BT2), as a template (Fig. 4b). Human SERCA is a known P-type ATPase with several isoforms differentially expressed in various tissues (regulating Ca^+2^ homeostasis) and an important drug target for cancer treatment^41,42^.

The transmembrane region of *Tg*SERCA contains 10 helices (M1–M10) embedded in the lipid bilayer (Fig. 4c). Structural superimposition of *Hs*SERCA2a and *Tg*SERCA revealed a close relationship (RMSD: ⁓5 Å) with maximum deviation in **A** and **N** domains and minimum deviation in transmembrane regions (Fig. 4d, e). A similar structural comparison with *Plasmodium falciparum* ATP6 (SERCA homolog) indicated a significant divergence (RMSD ⁓8 Å, Fig. S2a, b). We observed that the **A** domain of *Pf*ATP6 mainly consists of a loop region with maximum deviation. *Pf*ATP6 is suggested to be one of the artemisinin targets, yet its derivatives were found to be ineffective against *T. gondii*^43,44^, which may be due to structural deviation between *Tg*SERCA and *Pf*SERCA (Fig. S2b).

**Fig. 5:**
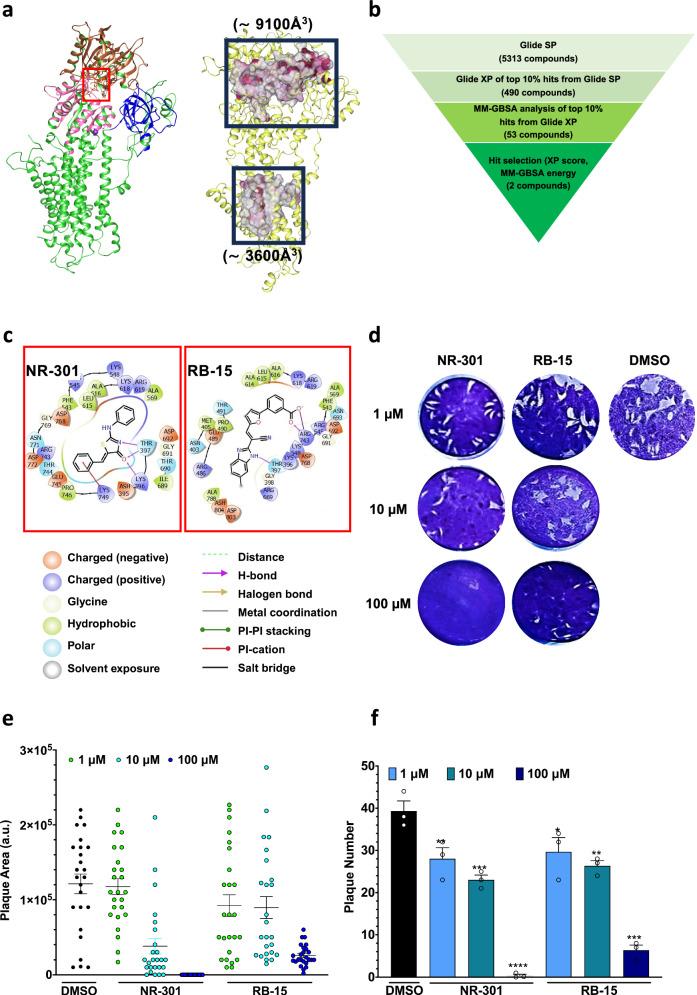
NR-301 and RB-15, binding to *Tg*SERCA, inhibit the lytic cycle of *T. gondii.* **a** The cavity analysis of *Tg*SERCA revealing two major cavities near the ATP and Ca^2+^ binding sites. **b** The docking-based screening to identify potential inhibitors inhibiting *Tg*SERCA. **c** Docking of NR-301 and RB-15 against *Tg*SERCA. **d**–**f** Plaques formed by tachyzoites cultured in the absence or presence of different concentrations of NR-301 and RB-15. Crystal violet-stained images illustrate the impact of inhibitors (1, 10, and 100 μM) or DMSO (control) on the lytic cycles in HFF monolayers. Quantification from *n* = 3 assays of the plaque size (**e**) and plaque number (**f**) are shown in graphs (means ± S.E.).

Evidence shows that human SERCA harbors at least two inhibitor-binding pockets, one near the ATP-binding site^42,45,46^. The *Tg*SERCA ATP-binding pocket is a part of the large cavity (9084 Å^3^) that spans three cytoplasmic domains: **A,** **P**, and **N** (largest cavity in *Tg*SERCA, Fig. 5a). The second inhibitor-binding pocket in human SERCA is near the Ca^2+^ binding channel^42^. This cavity in *Tg*SERCA (⁓3621 Å^3^) contains the transmembrane domain and is ⁓3x smaller than the first cavity (Fig. 5a). Notably, most SERCA inhibitors, including thapsigargin, bind selectively to the second pocket^42^. However, the ATP-binding site and its adjacent cavity have been recently exploited to design inhibitors of *Hs*SERCA^44,47^, prompting us to perform the following experiments.

### Computational docking identifies inhibitors of *Tg*SERCA and parasite growth

In light of the findings above, we set out to screen an in-house library of 5313 molecules to identify potential inhibitors of *Tg*SERCA and parasite growth (Fig. 5b). The docking protocol was initially validated by redocking the bound ATP to the binding site using the standard precision (SP) and extra precision (XP) modes in the Glide program. The library was first screened with Glide SP, and the top-scoring 490 hits (~10%) from this run were re-docked using Glide XP. The Molecular Mechanics with Generalized Born and Surface Area solvation (MM-GBSA) energy of the ~10% top-scoring hits from Glide XP (53 candidates) was determined using the Prime MM-GBSA method.

Two molecules, NR-301 and RB-15 were selected based on the RMSD between the docked poses generated by Glide SP and XP (RMSD ≤ 2.0 Å), hit-receptor interactions, and binding affinity assessed from the MM-GBSA energy (−54 and −46 Kcal/mol for NR-301 and RB-15, respectively). Further analysis of the binding pockets reveals that at least 25 residues were present at the NR-301 and RB-15 binding sites (for ATP binding, around 15 residues were involved), indicating a strong interaction with *Tg*SERCA (Fig. 5c). Lys396, Thr397, and Thr690 mainly mediated the ligand-receptor hydrogen bonds, while the *pi*-cation interactions primarily involved Lys749.

Comparative mapping of the ATP binding in *Hs*SERCA2a and *Tg*SERCA revealed high conservation (Fig. S3a, b). Hydrophobic (adenine region) and electrostatic (phosphate region) interactions stabilize the ATP binding in both proteins. NR-301 and RB-15 binding sites are positioned near ATP binding sites. We also noted similarities between human and *Toxoplasma* proteins for binding NR-301 and RB-15, but their molecular divergence supported the option for selective inhibition of *Tg*SERCA. We next evaluated the effect of both chemicals on the proliferation of human foreskin fibroblasts (host cells, Fig. S4) and the parasite growth in plaque assays (Fig. 5d–f, Fig. S5). None of the two chemicals impacted the host cell growth, but both inhibited the plaque formation in a dose-dependent manner. The parasite growth could be fully inhibited by 100 μM NR-301, while RB-15 significantly impaired the lytic cycle at this dose (Fig. S5). We estimated an EC_50_ value of ~2.4 μM for NR-301 and ~9.0 μM for RB-15.

### RB-15 and NR-301 directly impact the parasite, phenocopying the *Tg*SERCA mutant

**Fig. 6:**
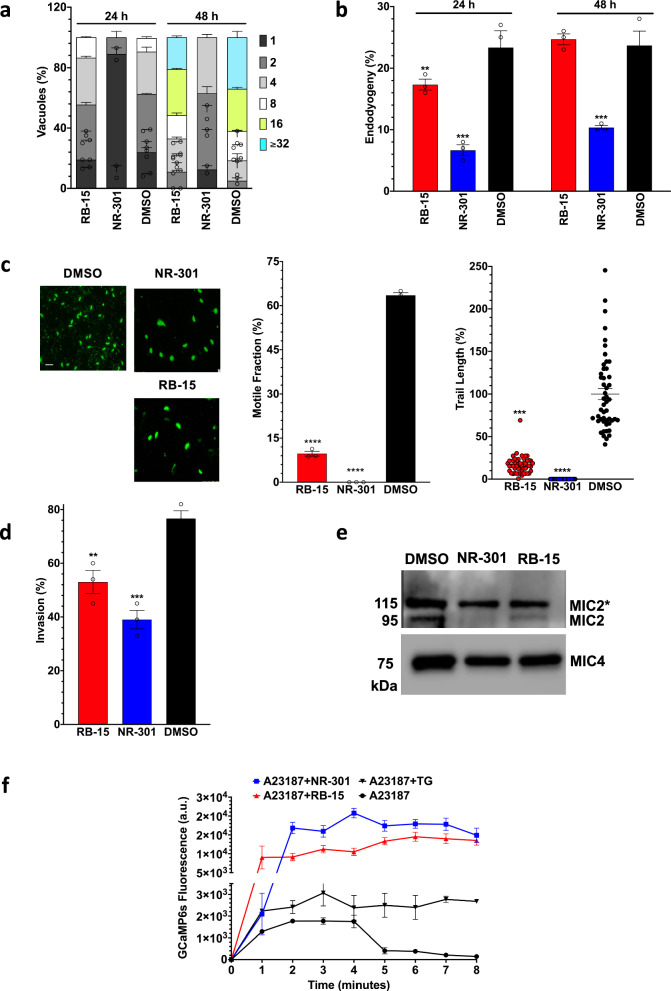
NR-301 and RB-15 inhibit the replication, motility and invasion, and impair calcium homeostasis in tachyzoites. **a**, **b** The proliferation and endodyogeny rates of tachyzoites cultured with NR-301 or RB-15 (100 μM) or DMSO. Parasites immunostained for *Tg*GAP45 (**a**) or *Tg*IMC3 (**b**) were evaluated. **c**, **d** Gliding motility and invasion efficiency of tachyzoites. The motile fraction and trail length were scored using *Tg*SAG1-stained parasites. The invasion rates were assessed by a two-color assay. The (**a**–**d**) show data from *n* = 3 assays (means ± S.E.). **e** Immunoblot of the parasite-secreted proteins (α*Tg*MIC2 and α*Tg*MIC4). Detection of pre-processed MIC proteins in the discharge is likely due to inadvertent lysis of tachyzoites. **f** Quantification of calcium in GCaMP6s-expressing parasites cultured in the presence of inhibitors (100 μM). The GFP signal was measured (relative fluorescence unit or RFU) and plotted after blank (no treatment) subtraction (*n* = 4 assays, means ± S.E.). Note that the graph includes the data points; however, they are not visible due to the large Y-axis. The original values used for this graph can be accessed in the Supplemental Data file.

The above finding encouraged us to examine the effect of RB-15 and NR-301 on individual lytic cycle events (Fig. 6). Both compounds inhibited intracellular replication, leading to a higher fraction of smaller PV than the DMSO control group (Fig. 6a). As observed above, the inhibitory impact of NR-301 on tachyzoite proliferation was more pronounced than RB-15. Equally, endodyogeny was disrupted in inhibitor-treated parasites; yet again, NR-301 showed a higher potency (Fig. 6b). Both chemicals inhibited the gliding motility and invasion of host cells by tachyzoites (Fig. 6c, d). The motile fraction and the trail length were significantly reduced (Fig. 6c). Inhibition of motility in the absence of host cells reveals a specific effect of these inhibitors on the parasite.

In the next step, the micronemal proteins and calcium homeostasis were investigated to decipher the mode of pharmacological action (Fig. 6e, f). As shown, both inhibitors impaired the discharge of mature MIC2 protein compared to the DMSO-treated control tachyzoites (Fig. 6e). We next tested the perturbation of calcium in parasites expressing GCaMP6s in the parasite cytosol (Fig. 6f), as reported previously^28^. Tachyzoites were induced by A23187 in the presence of NR-301, RB-15, or thapsigargin, and GCaMP6s fluorescence was recorded to gauge relative changes in cytosolic calcium. As expected, the ionophore increased the ion levels, which were subsequently restored to the basal levels. Inhibition of SERCA by thapsigargin resulted in sustained elevation of calcium. Noticeably, NR-301 or RB-15 further elevated the calcium level, triggering a 3-5x higher amount than thapsigargin (Fig. 6f). We conclude that inhibitors affect Ca^2+^ homeostasis and microneme secretion in tachyzoites, consequently impairing the lytic cycle.

### Ectopic expression of *Tg*SERCA restores the lytic cycle of a PtdThr Synthase mutant

**Fig. 7:**
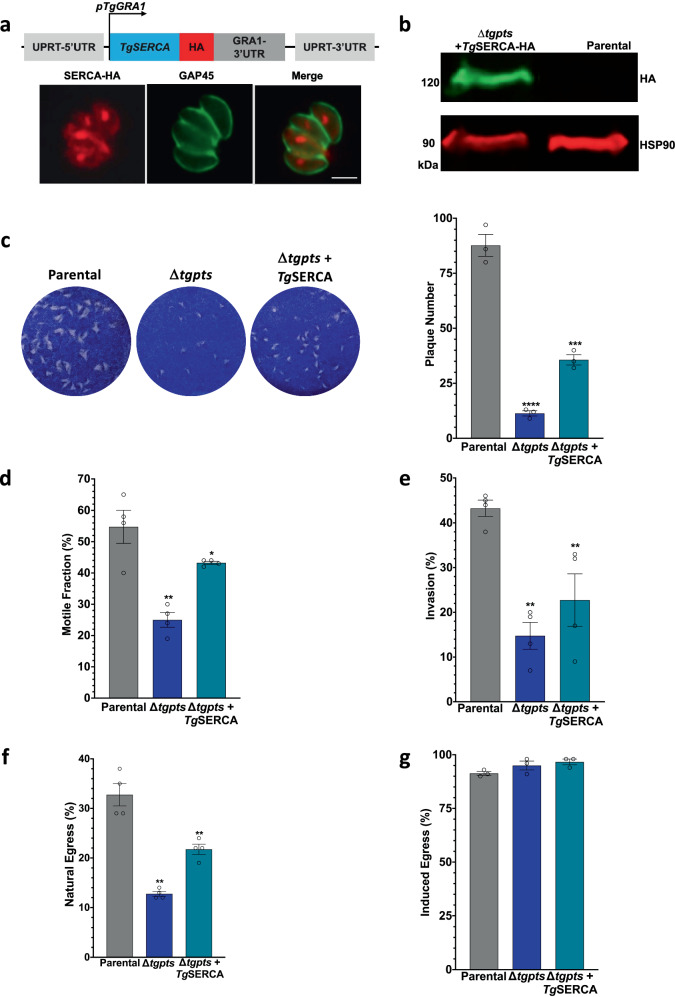
Ectopic expression of *Tg*SERCA can restore specific phenotypic defects in the Δ*tgpts* mutant. **a**, **b** Immunostaining confirming the expression of SERCA-HA in the Δ*tgpts* mutant (Scale, 5 μm). The SERCA expression cassette driven by the *Tg*GRA1 elements was inserted at the *UPRT* gene locus. **c** Plaques formed by the *RHΔku80Δhxgprt* (parental), Δ*tgpts,* and Δ*tgpts*-*Tg*SERCA strains (*n* =  3 experiments). **d**–**g** Motile fraction (30 min), invasion (60 min; MoI, 10), natural egress (MoI, 2; 48 h) and induced egress (MoI, 2; 24 h, 5 μM A23187) (*n* = 4 assays, means ± S.E.). The ectopic expression of SERCA-HA does not affect the replication and cell division (see Fig. S6).

Compositional aberrance of the ER membrane lipids is known to inhibit SERCA function and perturb the calcium homeostasis in mammalian cells^16^. Our previous work has reported a phosphatidylthreonine synthase mutant (Δtgpts) of *T. gondii* that lacks PtdThr but contains a proportionally elevated amount of otherwise much less abundant lipid, PtdSer (a natural homolog of PtdThr)^27^. PtdThr and PtdSer are synthesized in the parasite ER, the site of SERCA expression. PTS-null mutant is deficient in calcium storage and/or release and mostly phenocopies the SERCA-depleted strain. We, therefore, expressed *Tg*SERCA in the Δtgpts strain, anticipating a restoration of its lytic cycle (Fig. 7). The epitope-tagged protein was expressed in the mutant, as judged by immunofluorescence and immunoblot assays (Fig. 7a, b). Notably, all phenotypic defects, including plaques (Fig. 7c), gliding motility (Fig. 7d), parasite invasion (Fig. 7e), and natural egress (Fig. 7f), were partly reinstated. Further, egress was restored upon induction by A23187 (Fig. 7g). As shown previously^28^, the Δtgpts proliferated normally, and ectopic expression of *Tg*SERCA did not impact its intracellular replication (Fig. S6).

**Fig. 8:**
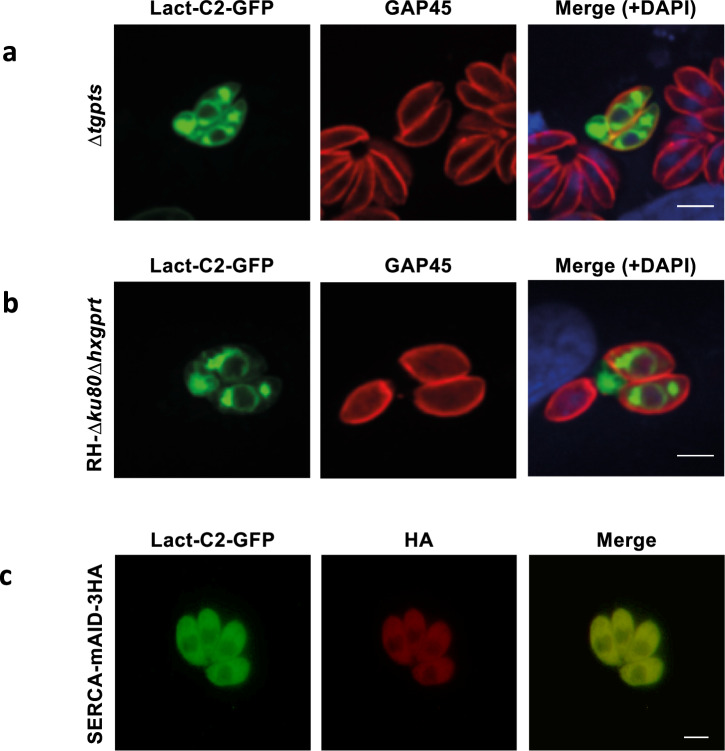
PtdSer and PtdThr are primarily present in the parasite ER, localizing with *Tg*SERCA. **a**–**c** Immunostained parasites of the Δ*tgpts* (PtdThr-null, PtdSer-enriched), *RH*Δ*ku80*Δ*hxgprt* (PtdThr-major, PtdSer-minor), and SERCA-mAID-3HA strains expressing Lact-C2-GFP. Parasites harboring the biosensor under the control of the *Tg*GRA1 elements were used to infect the host monolayer and subjected to immuno-fluorescent imaging (24 h post-infection) (Scale, 5 μm).

Following these findings, we were prompted to examine the co-localization of SERCA with PtdSer/PtdThr. We employed a biosensor of PtdSer, Lact-C2-GFP, consisting of the lipid-binding C2 domain of Lactadherin fused to GFP^48^. It was expressed in tachyzoites of the PTS mutant, which is deficient in PtdThr but proportionately enriched in PtdSer^27^. The protein was primarily localized in the perinuclear region, resembling the ER (Fig. 8a). Because the recently reported PtdSer synthase mutant is not deprived of PtdSer^32^, it was not feasible to unequivocally detect PtdThr using Lact-C2-GFP in this strain. Nonetheless, we transfected the parental strain, which expresses PtdThr as a major phospholipid (PtdSer is a minor lipid). Yet again, we detected a predominantly perinuclear expression of the biosensor, implying the presence of PtdThr in the ER (Fig. 8b). Not least, Lact-C2-GFP and SERCA-HA co-localized in tachyzoites (Fig. 8c). These results, taken together, signify the impairment of *Tg*SERCA in the PtdThr synthase mutant and a possible requirement of phospholipids for its optimal function in the parasite ER.

## Discussion

**Fig. 9:**
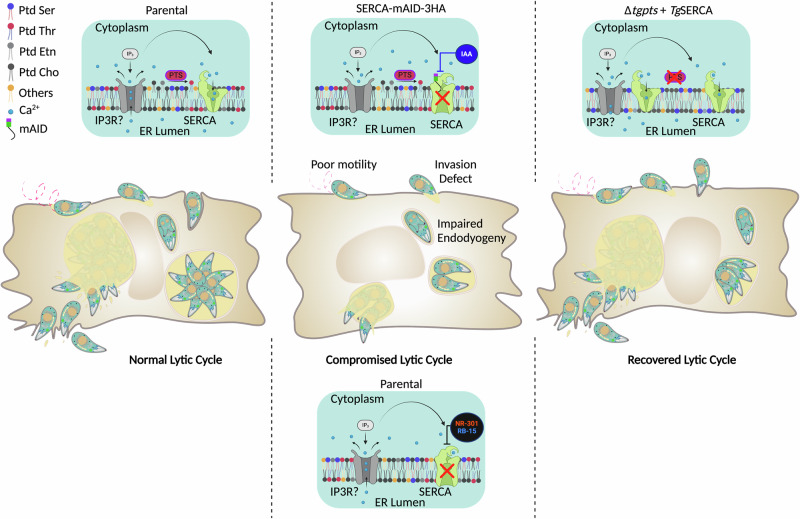
A model of SERCA function and its regulation during the lytic cycle of *T. gondii.* The illustration is based on this work and the literature cited herein. SERCA in the parasite ER membrane facilitates calcium refilling into the lumen after its IP3-induced discharge to the cytosol *via* an unknown ion channel (IP3?). Calcium is needed to activate the parasite locomotion and motility-dependent events (invasion, egress). IAA-mediated depletion of SERCA impairs the motility, invasion, replication, and egress of tachyzoites, culminating in a severely compromised lytic cycle. Potential inhibition of SERCA by newly-identified inhibitors (RB-15, NR-301) phenocopies SERCA-depleted mutant. On the other hand, ectopic expression of SERCA can restore the motility and associated defects in a PtdThr-null, PtdSer-enriched parasite strain with perturbed calcium homeostasis (Δ*tgpts*). The image was generated using the BioRender program (www.biorender.com).

This study shows SERCA as an essential *lipid-assisted* protein in the tachyzoite stage of *T. gondii* (Fig. 9). Using a conditional mutant of *Tg*SERCA, we disclose its essentiality for the lytic cycle of tachyzoites. Deploying computational modeling and molecule screening, our work identified two inhibitors of the lytic cycle. Finally, *Tg*SERCA-enabled restoration of the PtdThr synthase mutant offers a new premise for *lipid-mediated* regulation of calcium homeostasis in the endoplasmic reticulum of *T. gondii* (Fig. 9).

Calcium is a vital secondary messenger, regulating a range of functions in mammalian cells, such as cell cycle, signaling, contractility, metabolism, transcription, protein folding, apoptosis, and locomotion^14,49,50^. SERCA plays a significant role in refilling calcium stores in the endoplasmic reticulum^51–53^. Thapsigargin, a potent inhibitor of mammalian SERCA, can also inhibit its orthologs and perturb calcium homeostasis in *P. falciparum* and *T. gondii*^9,54^. While thapsigargin blocks the calcium tunnel, we identified the ATP-binding pocket of *Tg*SERCA as a potential target site for RB-15 and NR-301. Albeit not as potent, dysregulation of the parasite calcium, microneme discharge and gliding motility implies a specific effect of RB-15 and NR-301. Nonetheless, non-specific inhibition of the parasite cannot be excluded. Functional assays using purified *Tg*SERCA and synthesis of potent derivatives of these inhibitors can facilitate the search for more effective anti-infectives.

Using a calcium biosensor, we show that the SERCA-mAID-3HA strain is defective in refilling the cytosolic calcium into ER (Fig. 9). While this work has been revised, a parallel and independent preprint study has reported *Tg*SERCA’s role in facilitating interorganelle calcium homeostasis^55^, involving the endoplasmic reticulum, mitochondrion, and plant-like vacuole. The authors show that *Tg*SERCA helps refill cytosolic calcium into the ER and redistribute milieu-derived calcium ions into other organelles. *Tg*SERCA thereby assists in maintaining defined calcium levels in the cytosol (50–150 nM)^56^ and ER (>500 nM)^55^. Calcium release from its intracellular stores to the parasite cytosol is known to induce micronemal secretion^4^, and we show that SERCA enables a regulated secretion of MIC proteins, of which MIC2, in particular, supports gliding motility and host-cell invasion^4,39,57^. Once intracellular, SERCA promotes the proliferation of tachyzoites.

Zwitterionic and anionic phospholipids can influence the activity of mammalian SERCA in the ER^16^. Lipid reconstitution of enriched SERCA protein preparation from the rabbit skeletal muscle cells showed that its activity declines in the presence of PtdSer^17^. The protein is suggested to dimerize in the PtdSer-rich bilayer, and as a consequence, its catalytic site is rendered inaccessible to Mg^2+^-ATP. Besides, anionic phospholipids like PtdSer can restrict calcium slippage into the cytosol by SERCA, thereby increasing the calcium capture by the ER^27,58^. PtdThr is a natural homolog of otherwise-universal PtdSer, expressed in selected coccidian parasites^27,29,33^. The Δtgpts strain lacks PtdThr but has 3x higher level of PtdSer, which may adversely impact *Tg*SERCA function. In conclusion, this work provides new insight into structure-function features, physiological importance, regulation, and therapeutic potential of SERCA in *T. gondii*. Future research involving the reconstitution of recombinant SERCA in liposomes mirroring the membrane composition of the PTS-knockout and parental strains will provide additional insight.

## Materials and methods

### Biological reagents and resources

The RHΔ*ku80*Δ*hxgprt*^59^ and RHΔ*ku80*Δ*hxgprt*-TIR1^34,60^ strains were offered by Vern Carruthers (University of Michigan, Ann Arbor, MI) and David Sibley (Washington State University, St. Louis, MO), respectively. The Δtgpts mutant was generated in our previous work^27,33^. Lact-C2-GFP (*aka* GFP-Lact-C2) was provided by Sergio Grinstein (SickKids, Toronto, Canada). GCaMP6s construct was obtained from Loren Looger (Howard Hughes Medical Institute, Ashburn, USA). Antibodies recognizing *Tg*HSP90, *Tg*GAP45, and *Tg*IMC3 were donated by Sergio Angel (IIB-INTECH, Buenos Aires, Argentina), Dominique Soldati-Favre (University of Geneva, Switzerland), and Marc-Jan Gubbels (Boston College, Chestnut Hill, MA), respectively. Anti-*Tg*MIC2 and anti-*Tg*MIC4 antibodies were provided by Bang Shen (Huazhong Agricultural University, Wuhan, China). α*Tg*SAG1, αGFP, and αHA antibodies were purchased from Life Technologies and MBL International (Germany). The secondary antibodies (Alexa Fluor 488/594) were procured from Invitrogen (Germany), and immunoblot dyes (IRDye 680RD, IRDye 800RD) were from LI-COR Biosciences (Germany). Oligonucleotides for molecular cloning and transgenic work (Table S1) were obtained from Life Technologies.

### Parasite and host cell culture

Human foreskin fibroblast (HFF) cells were cultured based on a previous study^20^. In brief, cells were cultured in Dulbecco’s Modified Eagle Medium (DMEM) containing glucose (4.5 g/L) and supplemented with 1 mM sodium pyruvate, 2 mM glutamine, 100 μM MEM-nonessential amino acids, 100 U/mL penicillin, 100 μg/mL streptomycin, and 10% fetal calf serum. The cultures were maintained in a humidified incubator (37 °C, 5% CO_2_), grown to confluence, regularly harvested by trypsinization, and seeded into dishes, plates, or flasks. Tachyzoites were propagated by infecting confluent host cell monolayers (multiplicity of infection, 2-3) and passaged on alternate days. For phenotypic assays, parasitized host cells were scraped after 40-48 h post-infection and squirted through 22-gauge syringes to release intracellular tachyzoites. All biochemical analyses deployed fresh parasites washed by phosphate-buffered saline (400 *g*, 10 min, 4 °C) to remove cell debris. Parasites were counted and used directly or stored at -80°C for later usage.

### Engineering of transgenic parasites

The SERCA-mAID-3HA strain was generated by transfecting the RHΔ*ku80*Δ*hxgprt*-TIR1 strain with a CRISPR construct expressing Cas9 and gene-specific *sg*RNA (*pSAG1-Cas9-U6-sgSERCA*) along with a homology donor amplicon. The amplicon consisted of the mAID-3HA and HXGPRT selection cassette flanked by 40 bp of 5′ and 3′ homology arms designed for crossovers at the SERCA locus. It was amplified from the *pTUB1-YFP-mAID-3HA-HXGPRT* plasmid template. The sequence coding for SERCA-3′UTR-specific *sg*RNA was cloned by replacing *sg*UPRT in the *pSAG1-Cas9-U6-sgUPRT* vector (Q5 site-directed mutagenesis, New England Biolabs).

Tachyzoites (~10^7^) mixed with DNA (CRISPR plasmid: 20 μg, Amplicon: 14 μg) in filter-sterile Cytomix (120 mM KCl, 0.15 mM CaCl_2_, 10 mM K_2_HPO_4_/KH_2_PO_4_, 25 mM HEPES, 2 mM EGTA, 5 mM MgCl_2_ supplemented with 5 mM glutathione and 5 mM ATP; pH 7.6) were electroporated using a BTX instrument (2 kV, 50 Ω, 25 μF, 250 μs). Transgenic parasites were selected in a medium containing mycophenolic acid (25 μg/mL) and xanthine (50 μg/mL)^61^. Tachyzoites surviving the drug selection were isolated by limiting dilution, and individual clones were screened by genomic PCR for the 5’ and 3’ crossover events at the SERCA locus (primers in Table S1). Clones were further examined for the expression and regulation of SERCA-mAID-3HA by immunostaining. The eventual mutant expressed SERCA under the control of the native promoter in an IAA-dependent manner.

### Ectopic expression of SERCA and biosensors

For cloning the full-length open reading frame of *Tg*SERCA, total RNA was isolated from tachyzoites and reverse transcribed into first-strand cDNA (Life Technologies, Germany). *Tg*SERCA was cloned into *pTgGRA1-UPKO* plasmid (Table S1), enabling its expression under the regulatory elements of *Tg*GRA1. Similarly, the open reading frames of Lact-C2-GFP and GCaMP6s were cloned into the *pTgGRA1-UPKO* or *pTgSAG1-UPKO* plasmids (Table S1). Tachyzoites (1 × 10⁷) of the indicated strains were transfected with 10 µg of the respective plasmid. For stable expression, the ectopic expression cassettes were targeted at the uracil phosphoribosyltransferase (UPRT) locus. Tachyzoites with a disrupted UPRT locus were selected using 5’-fluorodeoxyuridine (FUdR, 5 μM)^62^. For transient expression, transfected parasites were grown in confluent host cell monolayers on glass coverslips and fixed by paraformaldehyde (24–36 h post-infection) for immunofluorescence staining.

### Indirect immunofluorescence assays

Confluent HFF cells grown on glass coverslips were infected with tachyzoites. Parasitized cells were fixed with 4% paraformaldehyde (15 min) and neutralized by 0.1 M glycine-PBS solution. Cells were permeabilized in 0.2% Triton X-100/PBS, followed by blocking with 2% bovine serum albumin (dissolved in 0.2% Triton X-100/PBS). Samples were incubated with primary antibodies (αHA, 1:3000; αGFP, 1:5000; α*Tg*GAP45, 1:3000; α*Tg*SAG1, 1:1000; α*Tg*MIC3, 1:2000) for 1 h and then washed 3x with PBS. Cells were treated with Alexa488- and 594-conjugated antibodies for 1 h, followed by 3x PBS washing, Fluoromount G/DAPI mounting (Southern Biotech, USA) and fluorescent imaging (Zeiss, Germany).

### Cell proliferation (scratch) assay

HFF cells were seeded in a 6-well plate. The confluent monolayer was scratched in the center of each well using a 200 μL pipette tip. The wells were washed twice with PBS to remove cellular debris, followed by the addition of media containing RB-15 or NR-301 (100 μM). DMSO was used as a vehicle control. Images of each well were captured using a microscope fitted with a MegCam camera (Olympus, USA).

### Lytic cycle assays

Standard methods were employed to determine the impact of SERCA depletion on the lytic cycle of tachyzoites (±500 μM IAA), as published elsewhere^18,19^. Parasites were pre-treated with IAA for 18–24 h before setting up the assay, and the auxin exposure was maintained during the experiment, as indicated in pertinent figure legends.

Plaque assays were performed using HFF monolayers seeded in 6- or 12-well plates. Host cells were infected with 100–200 parasites/well and incubated for 7 days without perturbation (±500 μM IAA). Samples were fixed with ice-cold methanol (10 min), stained with crystal violet (15 min) and washed 3x with PBS. Plaques were imaged by a light microscope and scored for size and numbers using the ImageJ software.

To determine the cell division, host cells on coverslips were infected (MoI, 1 for 24 h/48 h), fixed with 4% paraformaldehyde and immunostained using α*Tg*GAP45 (replication) or α*Tg*IMC3 (endodyogeny) antibodies. The PV were analyzed for the number of developing parasites to assess the replication phenotype. Endodyogeny was scored by calculating the fraction of tachyzoites with IMC3-stained daughter cells.

For quantifying the invasion efficiency, cells were infected with parasites (MoI, 10; 60 min), fixed in 4% paraformaldehyde (15 min) and then stained by α*Tg*SAG1 antibody. Samples were washed 3x with PBS and permeabilized by 0.2% Triton X-100 in PBS (15 min). They were immunostained by α*Tg*GAP45 and secondary antibodies (Alexa Fluor 488/594), followed by coverslip mounting in Fluoromount G and DAPI solution. Differential staining of *Tg*SAG1 and *Tg*GAP45 proteins distinguished extracellular and intracellular parasites. Those stained with α*Tg*GAP45 only were counted as invaded parasites. Egress assay involved a similar staining process, but HFFs were infected at MoI of 2 for 48 h (natural egress) or 24 h (A23187-induced egress). Tachyzoites stained with α*Tg*SAG1 only were enumerated to score the egress phenotype.

### Gliding motility assay

A sterile 24-well plate containing coverslips was coated with 300 µL of 0.01% BSA in PBS overnight (4°C). Fresh syringe-released tachyzoites (2 × 10^5^) were re-suspended in 300 µL of HBSS buffer (without calcium and magnesium) along with indicated inhibitors (NR-301 or RB-15) or DMSO (carrier solvent). Parasites were allowed to settle and glide (30 min, 37 °C), the supernatant was carefully removed, and then tachyzoites were fixed with 200 µL of 4% PFA (15 min, room temperature). Neutralization was achieved with 500 µL of 0.1 M glycine-PBS for 5 min, followed by blocking with 500 µL of 3% BSA-PBS for 20 min. Parasites were stained with mouse anti-*Toxoplasma* antibody in 3% BSA-PBS for 1 h, followed by 3x washing with PBS. Staining was performed with α*Tg*SAG1 and Alexa488 antibodies suspended in 3% BSA-PBS. Tachyzoites were imaged, and the motile fraction and trail length were quantified using the ImageJ software.

### Microneme secretion

Tachyzoites were suspended in DMEM containing 44 mM sodium bicarbonate, 20 mM HEPES, 2 mM glutamine, 10 µg/mL gentamicin, and 3% fetal bovine serum. After washing twice with IC buffer (5 mM NaCl, 142 mM KCl, 1 mM MgCl₂, 2 mM EGTA, 5.6 mM glucose, 25 mM HEPES, pH 7.2), parasites were centrifuged (400 × *g*, 10 min). The resulting pellet was resuspended in 500 µL of culture medium (2% FBS) supplemented with 1% ethanol. Tachyzoites were incubated in a water bath to allow secretion (37 °C, 30 min) and then pelleted twice (400 *g*, 10 min). Proteins in the supernatant were resolved using 8% SDS-PAGE and blotted onto PVDF membranes. Immunostaining was performed using rabbit α*Tg*MIC2 (1:10000), mouse α*Tg*MIC4 (1:10000) and horseradish peroxidase-conjugated (1:10000) antibodies. The protein bands were visualized by chemiluminescence (Azure Biosystems, USA).

### Calcium measurement

Transgenic tachyzoites (RHΔ*ku80*Δ*hxgprt* strain) expressing GCaMP6s were generated as reported previously^28^. Extracellular parasites were used to measure relative changes in the cytosolic calcium ions upon treatment with ionophore (5 μM A23187) in the presence of thapsigargin (1 μM), RB-15 or NR-301 (100 μM). Parasites were released from host cells and suspended in Ringer’s buffer (155 mM NaCl, 3 mM KCl, 2 mM CaCl₂, 1 mM MgCl₂, 3 mM NaH₂PO₄, 10 mM HEPES, pH 7.3) supplemented with 1 mM EGTA and 10 mM glucose. The assay was performed in a 96-well plate, with a final reaction volume of 100 μL, containing 40,000 parasites/well. Thapsigargin and DMSO-treated parasites served as the positive and negative control, respectively. Fluorescence was measured (excitation, 485 nm; emission, 525 nm), and the negative control values were subtracted from those of the experimental samples to normalize the data.

### Modeling of SERCA proteins

Domain analysis of *Tg*SERCA was achieved with InterPro (www.ebi.ac.uk/interpro). The *Tg*SERCA and *Pf*SERCA models were generated by homology modeling using Modeler version 10.1^63^. The sequences of both proteins were retrieved from UniProtKB [accession ID: Q5IH90 (*Tg*) and Q76NN8 (*Pf*)], and the structural homologs were identified by BLASTp against the Protein Data Bank (PDB)^64,65^. The PDB structure 7BT2, comprising the SERCA2a protein (E2 conformation) of *Homo sapiens* bound to ATP, was identified as the closest structural homolog of *Tg*SERCA and *Pf*SERCA (*Pf*ATP6). The *e*-value and query coverage were 0 and 93%, respectively^66^. The modeled structures were prepared using the Protein Preparation Wizard application of Schrödinger^67^. After a pre-processing step, the optimum protonation states of the hydroxyl groups and the Asn, His, and Gln residues at pH 7.0 were determined using the PROPKA program^68^. The structures were minimized using the OPLS4 force field, and the heavy atoms converged at an RMSD of 0.3 Å.

Structural superimpositions of *Hs*SERCA2a, *Tg*SERCA, and *Pf*SERCA were carried out using the pymol suite (www.pymol.org/2/). The lipid bilayer embedding *Tg*SERCA model was generated in the CHARMM-GUI server (www.charmm-gui.org, default parameters). Cavity analysis of *Tg*SERCA was performed using the CB-Dock2 server (www.cadd.labshare.cn/cb-dock2/index.php). Binding site analyses were executed using PLIP and PDBsum (www.ebi.ac.uk/thornton-srv/databases/pdbsum/).

### Ligand docking with *Tg*SERCA

The compound library was prepared using the LigPrep module in Schrödinger with the OPLS4 force field^69^. The ionization states of all compounds at pH 7.0 ± 2.0 were generated using Epik^70^. The original ionization states were also included. The compounds were desalted, and tautomers were not generated. The specific chirality was retained, and up to 32 structures were generated per compound. The final prepared library comprised 5315 compounds. The grid for molecular docking was prepared around the ATP binding site in the SERCA protein. The x, y, and z coordinates of the center of the grid were -39. 564, -60.645, and -19.382, respectively. The dimensions of the cubic grid were 26 Å along the x-, y-, and z-axes. The parameters were validated by redocking the bound ATP prepared using LigPrep. ATP was re-docked using Glide SP and XP. The RMSD values between the bound and docked poses were determined using Maestro and Schrödinger. RMSD values ≤ 2.0 Å between docked and bound poses suggest a good correlation between the docked conformation and native binding pose of ligands.

The in-house library of 5315 compounds was screened against the ATP binding site of *Tg*SERCA using the Glide SP^71,72^. Approximately 10% of the top-scoring hits from Glide SP were re-docked using the Glide XP algorithm^73^. The binding affinity of ~10% top-scoring hits from Glide XP was determined using the Prime MM-GBSA approach, Schrödinger. The MM-GBSA energy was calculated using the VSGB solvation model and the OPLS4 force field. The hit-receptor interactions were determined using Maestro and Schrödinger. Hit selection was achieved based on the Glide XP docking scores, Glide SP-XP RMSD of ≤2.0 Å, MM-GBSA energy, and hit-receptor interactions.

### Statistics and reproducibility

Unless specified otherwise, all graphs show the mean values with the standard error from multiple experiments (n = X assays/experiments; means ± S.E.). Statistical analysis was performed using the GraphPad Prism. Significance in experimental samples was tested against the control samples (e.g., parental, -IAA, DMSO) using Student’s *t*-test (*p ≤ 0.05; **p ≤ 0.01; ***p ≤ 0.001; ****p ≤ 0.0001). Images represent multiple assays.

### Reporting summary

Further information on research design is available in the Nature Portfolio Reporting Summary linked to this article.

## Supplementary information

Supplementary Information

Description of Additional Supplementary Files

Supplemental Data

Reporting Summary

## Data Availability

The data generated and/or analyzed during this study are provided in the main paper (Figs. 1–9) and supplementary files (Supplemental Figs. 1–6). The original gel and blot images are shown in the Supplemental Fig. 7. The source data for the graphs and images can be found in the Supplemental Excel files (Supplemental Data 1). Oligonucleotide sequences used for transgenic work are reported in Table S1. All resources are also available from the authors upon reasonable request.
