# Supplementary material for: Sarcoendoplasmic reticulum calcium ATPase is an essential and druggable lipid-dependent ion pump in Toxoplasma gondii
Source: Commun Biol. 2025 May 6;8:702. doi: 10.1038/s42003-025-08058-z (PMC12056192; doi:10.1038/s42003-025-08058-z)
Supplement: Supplementary file 1 — Supplementary Information [file 42003_2025_8058_MOESM1_ESM.pdf]

Figure S1

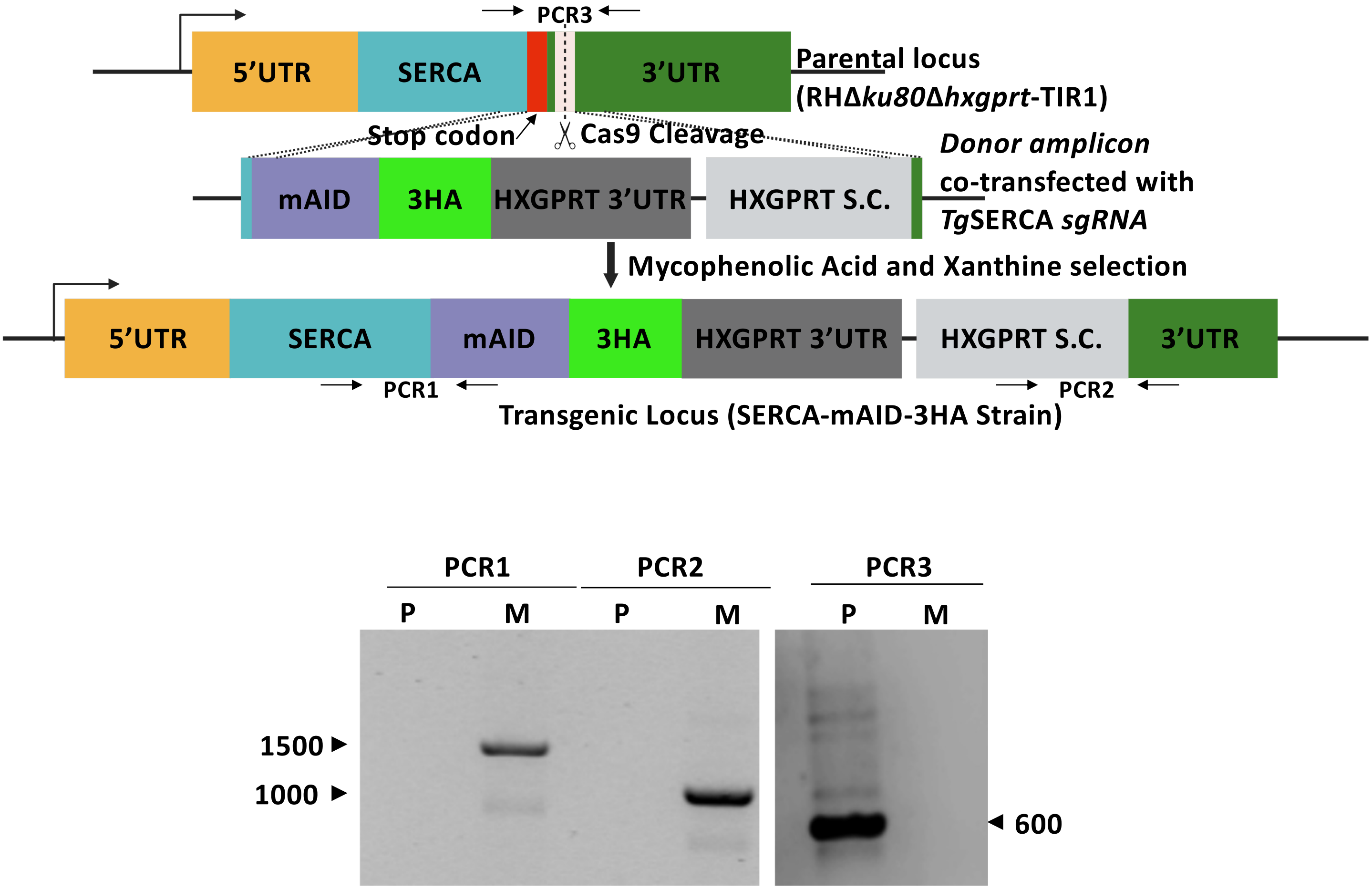

Genetic engineering of a SERCA-mAID-3HA mutant in *T. gondii* tachyzoites. Schematics of CRISPR/Cas9-assisted tagging of SERCA with mAID-3HA domain. A *pSAG1-Cas9-U6-sgSERCA* construct encoding for Cas9 nuclease and SERCA-3′UTR-specific *sg*RNA was transfected with a homology-directed donor sequence in tachyzoites. The donor amplicon was comprised of 5′ and 3′ recombination arms (40 bp each) flanking the mAID-3HA motif for 3′-tagging of SERCA and HXGPRT selection cassette. Parasites were drug-selected, and crossover-mediated genomic integration events were confirmed by recombination-specific genomic PCR. The eventual SERCA-mAID-3HA strain allowed the depletion of SERCA by Indole-3-acetic acid (IAA) (refer Fig 1).

Figure S2

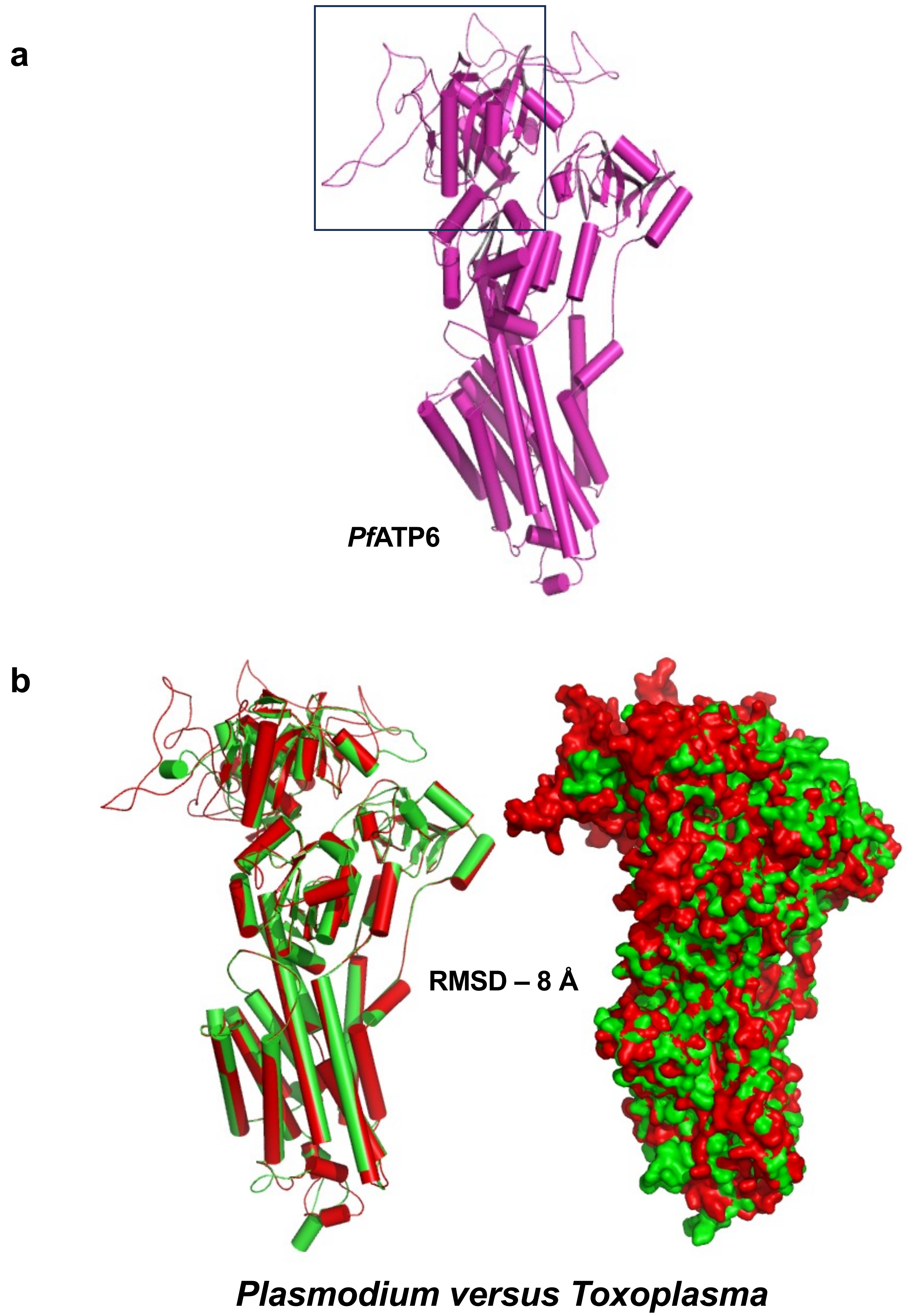

Structural comparison of *Tg*SERCA and *Pf*SERCA. (a) Homology-modeled *Pf*SERCA. **(b)** Superimposition of *Tg*SERCA (yellow) with *Pf*SERCA (red).

Figure S3

a

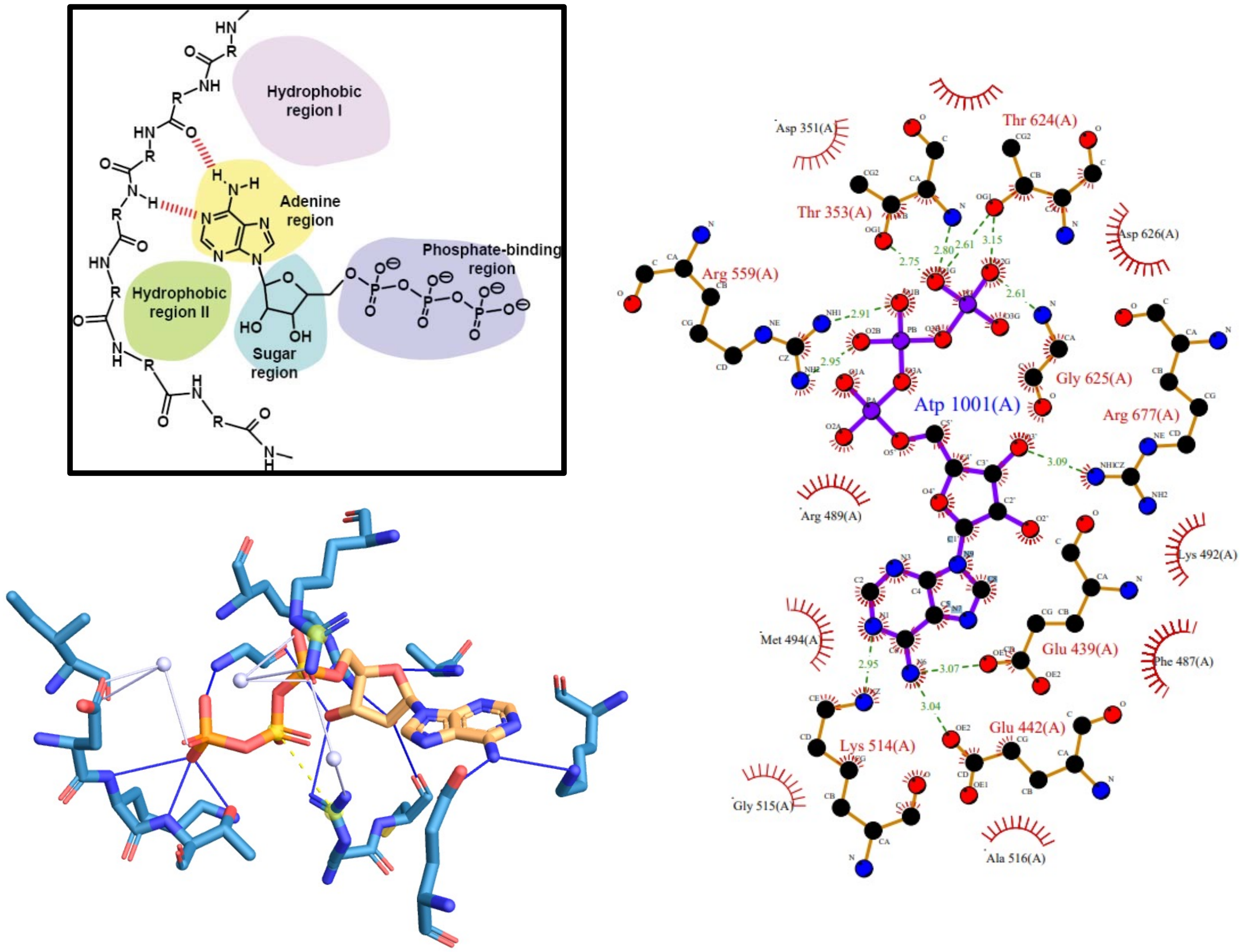

b

```
TgSERCA 548 ALTEGIRKKIQNDVDTMA--ADALRTLALAMKRDCGELADYDSASPSESRHPARKLLEDAANFAKIESDLIFLGLVGLMD
             +T G+++KI + +   +  +D LR LALA   +             E  H     LED+ANF K E++L F+G VG++D
HsSERCA 535 PMTSGVKQKIMSVIREWGSGSDTLRCLALATHDNPLRR---------EEMH-----LEDSANFIKYETNLTFVGCVGMLD

TgSERCA 626 PPRPEVSAAIDACRGAGIKVVMITGDNKLTAEAVASMIHIV-DDGCVGNCSFTGKEFEGLSLEEKKEVLSQDGVVFSRTE
            PPR EV++++  CR AGI+V+MITGDNK TA A+   I I   D  V + +FTG+EF+ L+   +++   +    F+R E
HsSERCA 601 PPRIEVASSVKLCRQAGIRVIMITGDNKGTAVAICRRIGIFGQDEDVTSKAFTGREFDELNPSAQRDACLNAR-CFARVE

TgSERCA 705 PKHKQMIIRLLRELGETTAMTGDGVNDAPALKQADIGVAMGIAGTEVAKEAADMVLADDNFSTIVAAVEEGRSIYNNMKA
            P HK  I+ +L+ + E TAMTGDGVNDAPALK+A+IG+AMG +GT VAK A++MVLADDNFSTIVAAVEEGR+IYNNMK
HsSERCA 680 PSHKSKIVEFLQSFDEITAMTGDGVNDAPALKKAEIGIAMG-SGTAVAKTASEMVLADDNFSTIVAAVEEGRAIYNNMKQ

TgSERCA 785 FIRYLISSNIGEVASIFFTAALGVPEGLSPVQLLWVNLVTDGPPATALGFNPPDLDVMKREPRHREDKLISNWIFLRYLL
            FIRYLISSN+GEV  IF+TAALG PE+L PVQLLWVNLVTDG PATALGFNPPDLD+M + PR+ ++ LIS W+F+RYL
HsSERCA 759 FIRYLISSNVGEVVCIFLTAALGFPEALIPVQLLWVNLVTDGLPATALGFNPPDLDIMNKPPRNPKEPLISGWLFFRYLA
```

The ATP binding sites in *Hs*SERCA2a and *Tg*SERCA. (a) The ATP binding site of *Hs*SERCA2a (PDB: 7BT2) displaying electrostatic and hydrophobic interactions as visualized by PLIP (*left*) and PDBsum (*right*) servers. **(b)** Sequence alignment of the human and *Toxoplasma* proteins to depict the conserved residues involved in ATP binding (black box). NR-301 and RB-15 binding sites are also marked (orange box).

Figure S4

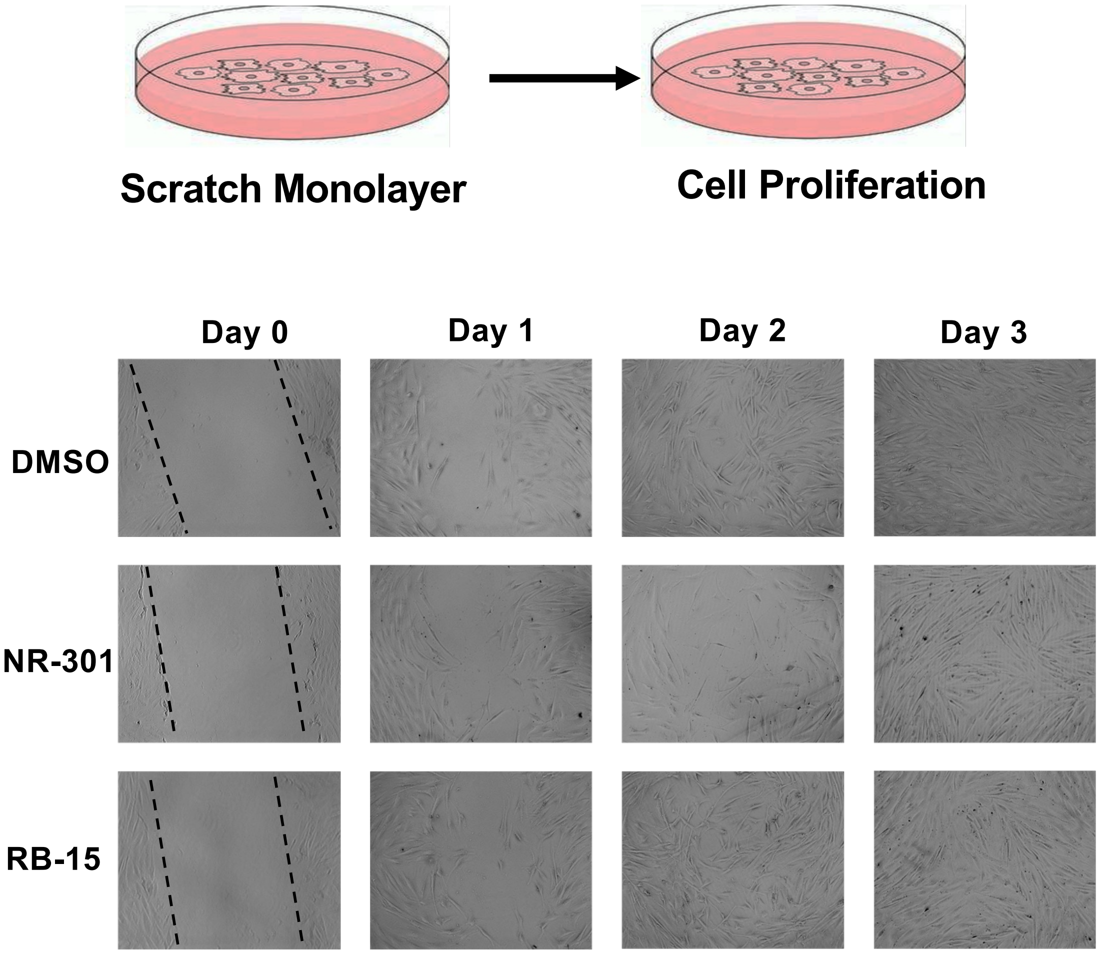

Growth of human foreskin fibroblasts is not impacted by NR-301 and RB-15. Cells were grown to confluence, followed by scratching with a 200 μL tip (dotted lines) and subsequent culture in the presence of specified inhibitors or DMSO (solvent vehicle). The schematics was generated using the bioRender program (www.biorender.com).

Figure S5

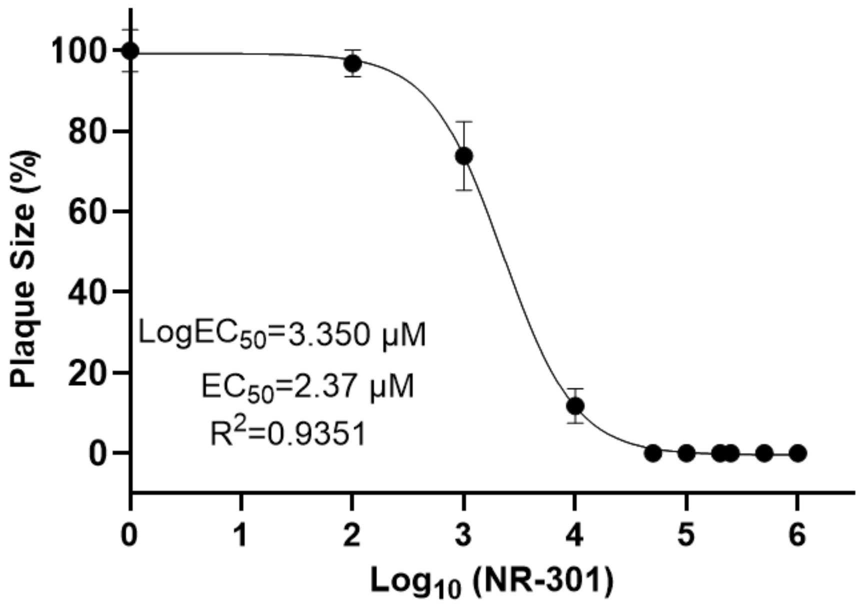

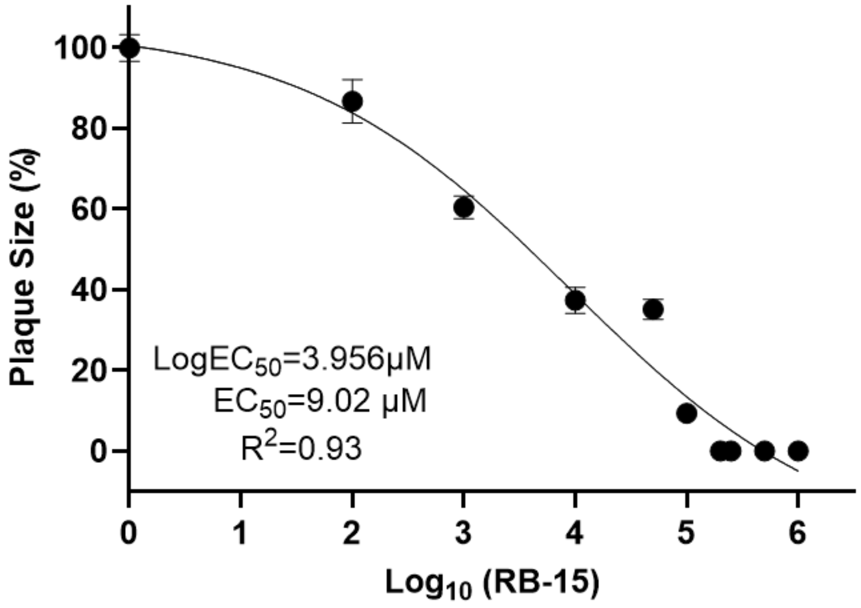

NR-301 and RB-15 inhibit the parasite growth in a dose-dependent manner. Plaques formed by tachyzoites in the presence of different concentrations of NR-301 and RB-15 were analyzed to calculate the EC_{50} values (n = 3 assays, means ± S.E.).

Figure S6

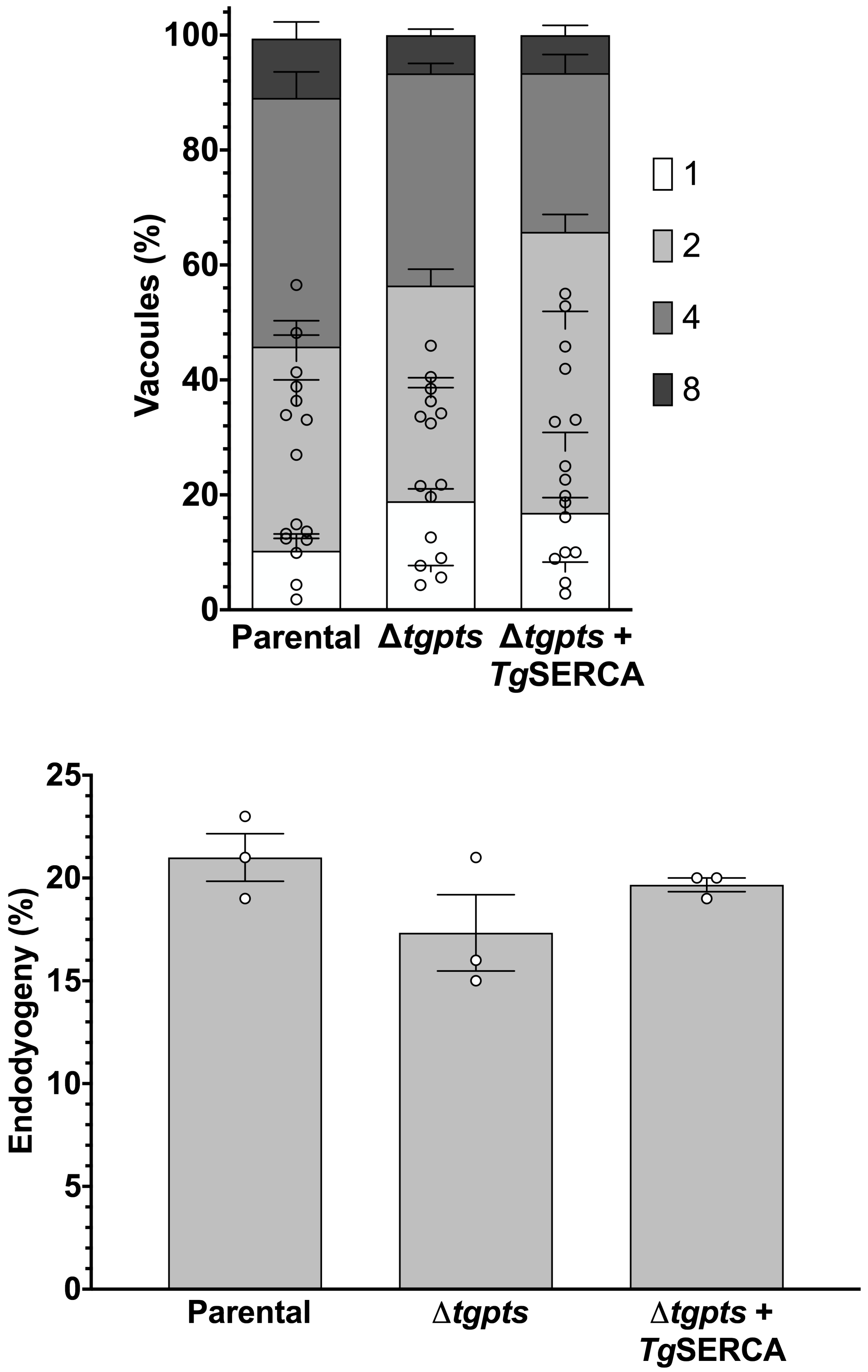

Expression of SERCA does not impact replication of the Δ*tgpts* mutant. The replication and endodyogeny assays performed with the *RHΔku80Δhxgprt* (parental), Δ*tgpts* and Δ*tgpts*-*Tg*SERCA strains. Intracellular tachyzoites immunostained for *Tg*GAP45 (*top panel*) and *Tg*IMC3 (*bottom panel*) were quantified (n= 3 assays, means ± S.E.). For other phenotypic assays, refer to Figure 7.

Figure S7: Uncropped Images

1c

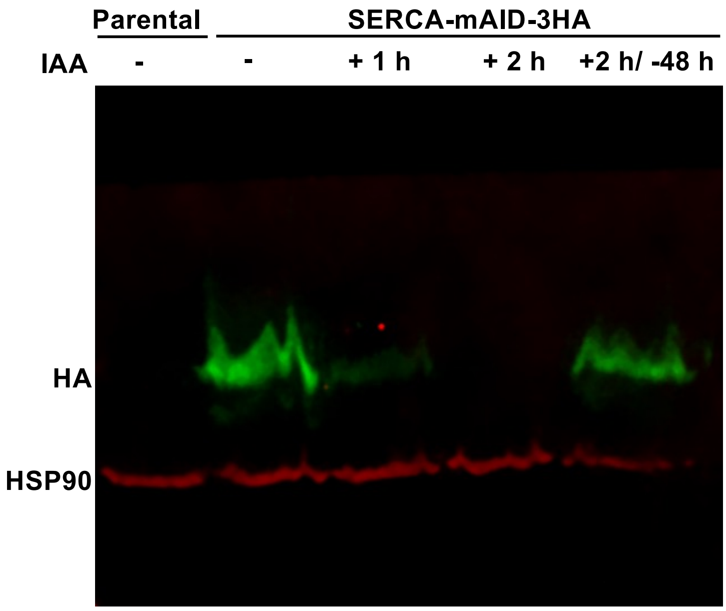

3c

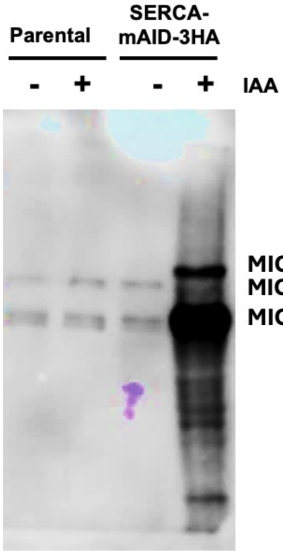

6e

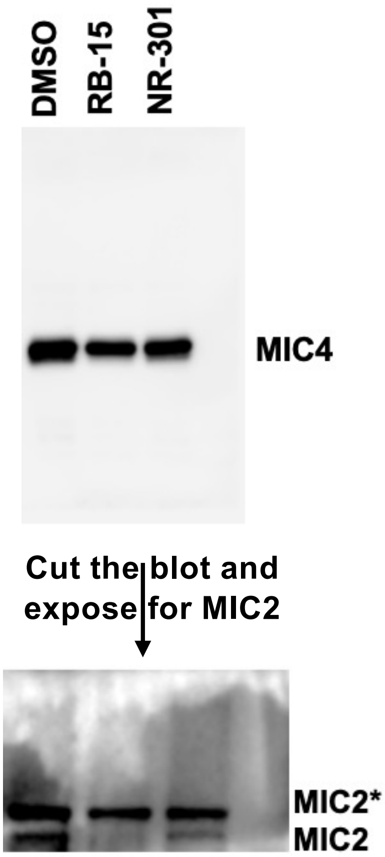

7b

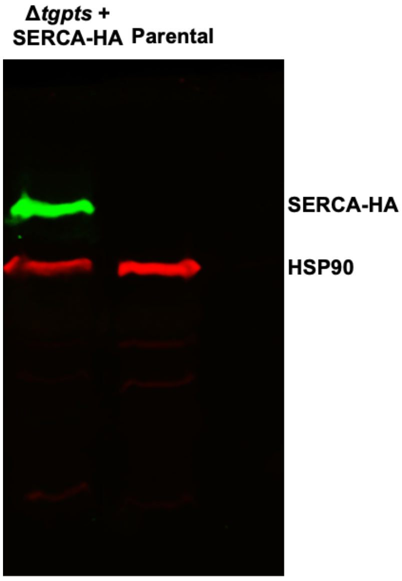

S1

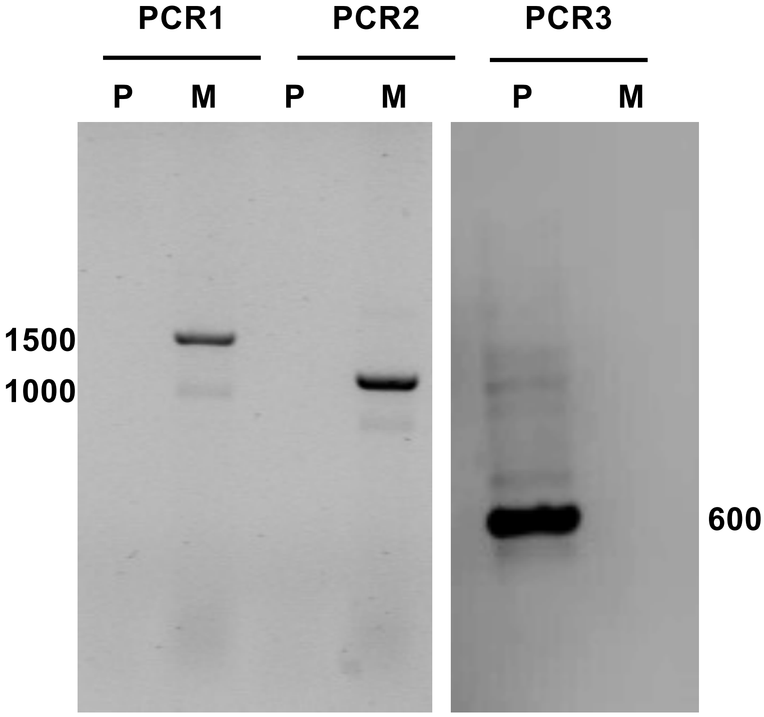

Table S1

Primer Name	Nucleotide Sequence 5'-3' (restriction site underlined)	Destination Vector and/or Objective
**Making of the SERCA-mAID-3HA mutant**		
SERCA-mAID-3HA-F	GAGCCTGTCGCGCTCCCAGTCGCAGCTGCGCAA GCTGCAGGAGAAGAGCGCGTGTCCTAA	*pTUB1-YFP-mAID-3HA-HXGPRT*
SERCA-mAID-3HA-R	GAGAGGCACGGCCTTTTCTGGGCCGTTCGGAG GCGAAAAAATAGGGCGAATTGGAGCTCC	
SERCA- gRNA-F	GGCCGTTCGGAGGCGAAAAAGTTTTAGAGCTAG AAATAGC	*pSAG1::CAS9-U6::sgUPRT*
CRISPR-R	GGCGTCTCGATTGTGAGAGC	
SERCA-Scr-PCR1-F	ACAAATCCATACCTCGTCGTC	Genomic screening of the *Tg*SERCA-mAID-3HA mutant
SERCA-Scr-PCR1-R	GTCTTTCTTCCTCCTATCGGAG	
SERCA-Scr-PCR2-F	ATTGAAGACGTCTGGATCGTTG	
SERCA-Scr-PCR2-R	GAGAGGCACGGCCTTTTCTG	
SERCA-Scr-PCR3-F	GTCGACGCTCAAGTGGCTTG	
SERCA-Scr-PCR3-R	TCTGAAGTGACTGTTCGGTGTCG	
**Expression of *Tg*SERCA in the Δ*tgpts* strain**		
SERCA-F (*Sbf*I)	CTCCCTGCAGGTGTCAAACGAGAAAGC CGTG	*pTgGRA1-UPKO* (expression of SERCA at the *UPRT locus*)
SERCA-R (*Pac*I)	CTCTTAATTAATTAAGCGTAATCTGGAACATCGTA TGGGTACTGCAGCTTGCGCAGCT	
**Expression of Lact-C2-GFP in the Δ*tgpts*, RHΔ*ku80*-Δ*hxgprt*, *Tg*SERCA-mAID-3HA strains**		
Lact-C2-GFP-F (*Nsi*I	CTCATCATGCATATGGTGAGCAAGGGCGAG	*pTgGRA1-UPKO* (transient expression of Lact-C2-GFP)
Lact-C2-GFP-F (*Pac*I)	CTCATCTTAATTAACTAACAGCCCAGCAGCTCC	
**Expression of GCaMP6s in the SERCA-mAID-3HA and RHΔ*ku80*-Δ*hxgprt* strains**		
GCaMP6s-F1	TCTGGAACATCGTATGGGTACTTCGCTGTCATCA TTTGTACAAAC	*pTgSAG1-UPKO* (expression of GCaMP6s at the *UPRT locus*)<br><br>(Gibson assembly)
GCaMP6s-R1	CGGTTGTATGGACAAAATGCATTCTCATCATCAT CATCATC	
GCaMP6s-F2	GTACAAATGATGACAGCGAAGTACCCATACGAT GTTCCAGATTACG	
GCaMP6s-R2	ATGATGATGATGAGAATGCATTTTGTCCATACAA CCGTGTGTTTACA	

Oligonucleotides used in this study.
